# Practical Notes on Diagnosis and Treatment

**Published:** 1910-08-20

**Authors:** 


### Practical Notes on Diagnosis and Treatment.

**Bronchitis in Typhoid.**

The bronchitis of enteric fever is characterised by a short cough, with little or no expectoration.—*Dr. William Hunter.*

**Carcinomatous Glands.**

Carcinomi upon the organs of generation is almost sure to follow the superficial lymphatics in front of the pubes where these interlace and cross, so that one must never omit to examine the glands in both groins.—*Mr. Lockwood.*

**Subinvolution.**

Besides ergot, an essential part of the treatment is the giving of purgatives, especially magnesium sulphate. An acid tonic with ergot and mag. sulph. generally answers particularly well. In advanced cases where there is induration of the uterus a little mercury is desirable.—*Dr. Amand Routh.*

**Belladonna in Broncho-pneumonia.**

Broncho-pneumonia is a very fatal disease in early life, and treatment is often unsatisfactory. Quarter-grain doses of the extract of belladonna will be found very useful. This dose should be given whatever the age of the child, and is to be repeated every three or four hours. Beyond slight delirium there are no toxic effects.—*Dr. J. A. Coutts.*

**Blood-letting in Hemiplegia.**

If you see the patient early, when the hæmorrhage is beginning, you may do good by bleeding; but bleeding, if it is to be of any use, must be free and continued till a definite fall in blood pressure is produced. I believe I have saved life by that means more than once, but it is no use bleeding unless you reach the case early.—*Dr. Samuel West.*

**Cancer of the Gall Bladder.**

The presence of gall-stones in cancerous gall bladders is probably as frequent as 95 per cent.; this does not necessarily mean that calculous cholecystitis is a pre-cancerous condition, but that the pathological conditions of the epithelium lining the gall bladder, which cause it to produce cholesteria, do increase its predisposition to cancer. Not only is it right to remove such calculi, but I would go a step further and urge that such unhealthy gall bladders should also be removed.—*Mr. Bland-Sutton.*

**Hæmoptysis.**

With consumptive patients under continuous observation it may sometimes be possible to observe some restlessness or other slight change of condition for a few hours or a day before an attack, which the experienced recognise as a warning of an impending hæmoptysis. These premonitory signs may be coincident with the actual hæmorrhage in the lungs, the blood being coughed up later. There is generally a fall in temperature immediately after the hæmorrhage, and there may be a rise before the attack, and another quickly after.—*Dr. Edward Squire.*

**Renal Typhoid.**

Cases have been reported in which pyrexia and hæmaturia were the sole symptoms of an attack of typhoid fever. Widal's test was positive, and the urine contained large numbers of the bacillus typhosus.

**Seborrhœic Dermatitis.**

In dealing with the earlier simply seborrhœic inflammation the most efficient remedy at our disposal is sulphur, either as ointment, lotion, or bath, or it may be prescribed internally.—*Dr. James Galloway.*

**Pessaries and Cancer.**

Cancer of the vagina is rare, but the use of pessaries is excessively common, so that, though the persistent use of a pessary is not favourable to cleanliness, it cannot be charged with causing cancer, although years may have elapsed without its being moved.—*Mr. Bland-Sutton.*

**Ergot in Uterine Myomata.**

It has been shown that the continuous administration of ergot by producing arterial contraction throws an increased strain on the cardiac muscle; this is the more harmful because in these women considerable anæmia is present as well. Hence old cases of " fibroids " who have been treated for years by medicinal measures exhibit cardiac dilatation, which is a serious drawback to the usually inevitable operation.—*Dr. Victor Bonney.*

**The Climacteric and Carcinoma Cervicis.**

It is a mistake to suppose that the menopause is normally associated with unusual loss of blood. It is a fatal mistake to administer ergot in cases of genital hæmorrhage without having taken the precaution of making first a digital examination to discover the cause. Numbers of inoperable cervical carcinomas are sent to gynæcologists with a history of having been treated with ergot for a long time without an examination having been made. If an examination is refused, the practitioner ought to decline to treat the case.—*Dr. T. W. Eden.*

**Appendicitis.**

Suppuration is almost universal in appendicitis; in cases recovering without the need of operation the pus is discharging almost always by the appendix; if the temperature is raised for as long as four days, pus is present, and the same may be concluded if there is a mass in the abdomen. If pus is present, it often discharges satisfactorily internally, about three times as often into the small as into the large bowel. If the discharge is adequate, then surgical intervention is unnecessary; but if there are symptoms of pent-up pus or an inadequately discharging pus formation in the appendix, then surgical intervention is required.—*Mr. Edred Corner.*

